# Reduced scan range abdominopelvic CT in patients with suspected acute appendicitis - impact on diagnostic accuracy and effective radiation dose

**DOI:** 10.1186/s12880-019-0304-x

**Published:** 2019-01-11

**Authors:** Dominik Zinsser, Michael Maurer, Phuong-Linh Do, Jakob Weiß, Mike Notohamiprodjo, Fabian Bamberg, Ahmed E. Othman

**Affiliations:** Department of Diagnostic and Interventional Radiology, Eberhard-Karls-University Tuebingen, University Hospital Tuebingen, Hoppe-Seyler-Str. 3, 72076 Tuebingen, Germany

**Keywords:** Appendicitis, Computed tomography, Scan range, Radiation dose

## Abstract

**Background:**

To evaluate a reduced range CT protocol in patients with suspected acute appendicitis as compared to standard abdominal CT regarding diagnostic performance, effective radiation dose and organ doses.

**Methods:**

In this study, we retrospectively included 90 patients (43 female, mean age 56.7 ± 17 years) with suspected acute appendicitis who underwent CT of abdomen and pelvis. From those CTs, we reconstructed images with a reduced scan range from L1 to the the pubic symphysis. Full range and reduced range datasets were assessed by two radiologists for i) coverage of the Appendix, ii) presence/absence of appendicitis and iii) presence of differential diagnoses. Furthermore, effective radiation doses as well as organ doses were calculated using a commercially available dose management platform (Radimetrics, Bayer HealthCare).

**Results:**

The Appendix was covered by the reduced range CT in all cases. In 66 patients CT confirmed the presence of appendicitis. In 14 patients, other relevant differential diagnoses were identified by CT, whereas in 10 patients no relevant findings were detected. Both readers identified all patients with appendicitis on both full and reduced range CT.

For reduced range CT, total effective dose was 39% lower than for full range CT (reduced range: 4.5 [1.9–11.2] vs. full range: 7.4 [3.3–18.8] mSv; *p* ≤ 0.001). Notably, a remarkable reduction of organ dose in the female breasts by 97% (0.1 [0.1–0.6] vs. 3.8 [0.5–18.8] mSv; *p* ≤ 0.001) and in the testicles in males by 81% (3.4 [0.7–32.7] vs. 17.6 [5.4–52.9] mSv; *p* ≤ 0.001) was observed for reduced range CT compared to full range CT.

**Conclusions:**

In patients with suspected acute appendicitis, reduced range abdominopelvic CT results in a comparable diagnostic performance with a remarkable reduction of total effective radiation dose and organ doses (especially breast dose in female and testicle dose in male patients) as compared to full range CT.

## Background

Appendicitis is a common cause of abdominal pain and affects patients of all age groups [1]. Despite the typical clinical presentation, imaging is crucial to rule out differential diagnoses, make the correct diagnosis and therefore prevent unnecessary surgery. While ultrasound is frequently used as the first-line imaging modality, it is operator and patient dependent, and therefore its results are often inconclusive. Computed tomography is a powerful and widely used alternative with high accuracy in diagnosing acute appendicitis as well as alternative diagnoses. However, its major drawback is the radiation exposure which is of interest especially in younger persons who are frequently affected by acute appendicitis [2]. Consequently, prior studies investigating the diagnostic value of low-dose CT with decreased tube current showed high diagnostic accuracy in patients with appendicitis [3–7].

As an alternative approach, several authors proposed a limited CT examination protocol with reduced scan range focused on the lower parts of the abdomen and pelvis, a method that has also been adopted to other body regions [8]. Whereas some studies concluded that a CT examination limited to the pelvis would be inappropriate in patients with suspected appendicitis because alternative diagnoses cannot reliably be ruled out [9, 10], several subsequent studies investigating wider scan ranges additionally including the lower parts of the upper abdomen reported satisfying results in adults as well as in children [11–14]. Compared to a conventional abdominopelvic CT examination, this approach is associated with a significantly reduced scan range. These works defined the scan range according to bony landmarks like the pubic symphysis and certain vertebral bodies which are usually well-recognizable on the CT localizer radiograph. According to several studies, a scan beginning at the upper border of the second lumbar vertebral body reliably depicts most of the renal collecting system and therefore is sufficient to display the appendix and to rule out urolithiasis as an important alternative diagnosis in these patients [12, 14]. However, possible diseases of the gall bladder like cholecystitis which may mimic appendicitis in rare cases would probably be missed. Furthermore, these previous studies focused on CT dose reductions in general and did not report organ doses at all or only for few selected organs like breasts and testicles. Therefore, the aim of our study was to evaluate the diagnostic value of a CT protocol with a slightly less limited scan range in the upper abdomen compared to precedent works from the first lumbar vertebral body to the pubic symphysis in patients with suspected acute appendicitis and to highlight the reduction of effective radiation dose as well as organ doses.

## Methods

### Patients

The local institutional review board approved this study. Requirement for informed consent was waived.

We retrospectively included consecutive patients referred to our department during a period of 14 months (January 2015 – February 2016) according to the following criteria: 1. Suspected appendicitis, 2. Inconclusive ultrasound (acute appendicitis was neither confirmed nor ruled out and no alternative diagnosis was established), 3. Available consecutive CT. Exclusion criteria was age under 16 years. Those patients were identified by searching our single institution radiology database for the keyword “appendicitis” in the radiology report.

### CT data acquisition

The examinations were conducted on four CT scanners, two of those were dual-energy (SOMATOM Force and SOMATOM Definition Flash) and two were single-energy scanners (SOMATOM Sensation 64 and SOMATOM Definition AS+, all Siemens Healthineers, Forchheim, Germany). Patients were placed supine and scanned in craniocaudal direction after acquisition of an anterior-posterior scout acquisition. In absence of contraindications, patients received a body-weight adapted volume (1.3 ml/kg body-weight) of intravenous contrast agent (Iopromid 370 mg/ml, Bayer Vital, Leverkusen, Germany, or Iomeprol 400 mg/ml, Bracco Imaging, Konstanz, Germany) through an antecubital vein catheter followed by a bolus of 30 ml saline with a flow rate of 2.2. ml/s. Imaging was initiated 90 s after contrast agent administration. Automated tube current modulation (CareDose 4D, Siemens Healthineers) was routinely activated. See Table 1 for details on scanning parameters.

**Table 1:** Scanning parameters

CT scanner	SOMATOM Force	SOMATOM Definition Flash	Sensation 64	SOMATOM Definition AS+
Pitch	0.6	0.7	0.9	0.6
Collimation	2 × 128 × 0.6 mm	2 × 128 × 0.6 mm	64 × 0.6 mm	128 × 0.6 mm
Tube voltage	100/150 kV Sn	100/140 kV Sn	120 kV	120 kV
Tube current	190/95 ref. mAs	196/151 ref. mAs	250 ref. mAs	220 ref. mAs

*kV* Kilovolts, *Sn* Tin filter, *ref mAs* Reference milliampere seconds

### Image reconstruction

**Fig. 1:**
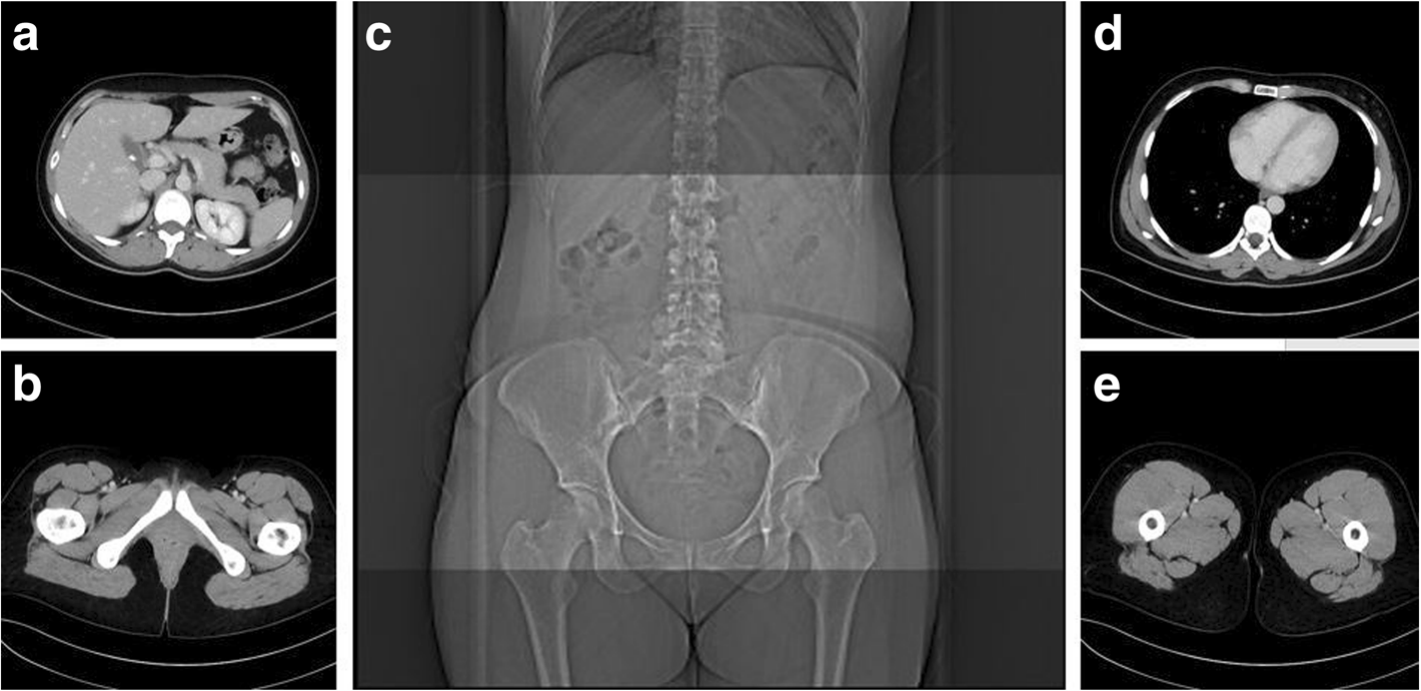
Axial reconstructed images displaying the upper and lower border of reduced range CT (**a** and **b**) as defined by the top of L1 and the pubic symphysis (**c**) compared to those of full range CT (**d** and **e**)

All examinations were axially reconstructed using a soft body convolution kernel (full range CT). Moreover, we reconstructed further axial series from the originally obtained image data with a limited range from the superior endplate of the first lumbar vertebra to the inferior edge of the pubic symphysis with the same slice thickness (virtual reduced range CT, Fig. 1). With both dual-energy scanners, iterative reconstruction algorithms were used (advanced modeled iterative reconstruction, ADMIRE, or sinogram affirmed iterative reconstruction, SAFIRE, both Siemens Healthineers), while the examinations performed using the single-energy scanners were reconstructed with filtered back projection.

### Radiation dose assessment

We assessed the radiation dose of the full range as well as the virtual reduced range CT in terms of whole body effective dose and organ doses. These were calculated using a commercially available dose monitoring and tracking software (Radimetrics, Bayer Healthcare, Leverkusen, Germany). Based on anthropomorphic phantoms and Monte Carlo simulations, this software provides effective dose and organ dose values of the original full range CT scan according to the weighting factors published in the International Commission on Radiation Protection 103 report. By using an interactive tool which allows to manually adjust the superior and inferior border of the scan range, we additionally obtained effective dose and organ dose values for the virtual reduced range CT in a second step. Besides the whole body effective dose, we recorded the dose values for the following organs: adrenals, colon, esophagus, gall bladder, heart, kidneys, liver, lungs, muscle, pancreas, red marrow, skeleton, skin, small intestine, spleen, stomach and urinary bladder. Furthermore, the organ dose of breasts, ovaries and uterus was recorded in female subjects as well as the testicle dose in males.

### Image analysis

Two radiologists determined if the appendix was entirely displayed by the virtual reduced range CT scan, evaluated the presence or absence of appendicitis according to commonly accepted criteria (diameter > 6 mm, wall thickening, periappendiceal fat stranding, periappendiceal free fluid, presence of appendicolith) [15] and recorded the presence of alternative diagnoses and incidental findings. In addition, the full range CT was also evaluated for the presence of differential diagnoses and incidental findings serving as reference standard. Any discrepancies were solved by consensus. As one purpose of reading was to identify additional findings displayed by full range CT that were not captured by reduced range CT, readers had access to the original radiology report if deemed necessary.

### Statistical analysis

For statistical analyses, MedCalc Statistical Software version 12.6.1.0 was used (MedCalc Software, Ostend, Belgium). The D’Agostino-Pearson normality test was performed to test for normal distribution of quantitative data. Since all samples did show non-normal distribution, data are represented by median with minimal and maximal values and were compared using the Wilcoxon signed-rank test. Results were considered significant at a level of *p* ≤ 0.05.

## Results

### Patients

In total, 90 patients were included (43 female, 47 male, mean age 56.7 ± 17 years). We did not exclude any patient as no abdominopelvic CT examinations of individuals under the age of 16 years with suspected acute appendicitis were conducted during the inclusion period. Eighty-three patients received intravenous contrast agent while an unenhanced examination was conducted in seven patients due to contraindications for intravenous contrast medium. The majority of 54 patients was examined using the SOMATOM Force while the remaining 36 patients were examined with the SOMATOM Definition Flash (24 patients), Sensation 64 (10 patients) and Definition AS+ (2 patients). When iterative reconstruction algorithms were available, ADMIRE level 2 was chosen for 37 examinations and ADMIRE level 3 for 17 examinations (SOMATOM Force), while SAFIRE level 2 was chosen for 23 and SAFIRE level 1 for one examination (SOMATOM Definition Flash).

### Radiation dose assessment

**Fig. 2:**
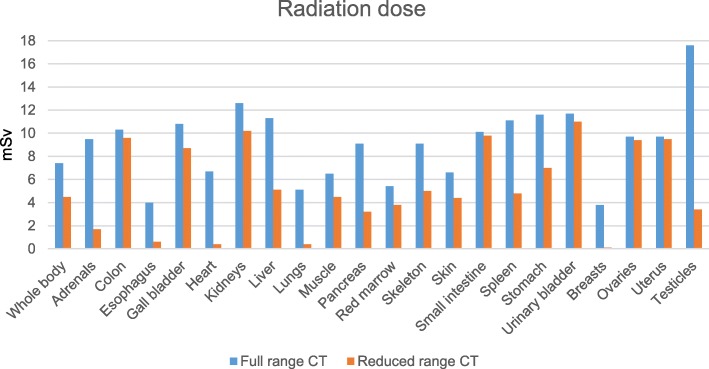
Comparison of median whole body dose and organ doses for full range CT and virtual reduced range CT

The whole body effective dose of the virtual reduced range CT was significantly lower compared to full range CT yielding a dose reduction of 39.2% (4.5 [1.9–11.2] vs. 7.4 [3.3–18.8] mSv, *p* ≤ 0.001). A remarkable dose reduction for the reduced range CT of 97.4 and 80.7%, respectively, was also observed for the breasts in female (0.1 [0.1–0.6] vs. 3.8 [0.5–18.8] mSv, *p* ≤ 0.001) as well as for the testicles in male patients (3.4 [0.7–32.7] vs. 17.6 [5.4–52.9] mSv, *p* ≤ 0.001). The detailed radiation doses are displayed in Table 2 and Fig. 2.

**Table 2:** Radiation dose

Organ	Full range CT (median [range], mSv)	Virtual reduced range CT (median [range], mSv)	Reduction (%)
Whole body	7.4 [3.3–18.8]	4.5 [1.9–11.2]	39.2
Adrenals	9.5 [4.9–21.6]	1.7 [0.6–12.8]	82.1
Colon	10.3 [5.1–23.5]	9.6 [4.9–22.1]	6.8
Esophagus	4.0 [1.5–9.7]	0.6 [0.2–4.7]	85.0
Gall bladder	10.8 [5.5–23.6]	8.7 [4.4–18.4]	19.4
Heart	6.7 [1.8–17.1]	0.4 [0.1–6.4]	94.0
Kidneys	12.6 [6.7–28.2]	10.2 [5.2–20.5]	19.0
Liver	11.3 [5.5–25.5]	5.1 [2.2–15.1]	54.9
Lungs	5.1 [1.4–13.5]	0.4 [0.1–5.1]	92.2
Muscle	6.5 [2.9–16.6]	4.5 [2.2–11.2]	30.8
Pancreas	9.1 [4.5–20.6]	3.2 [1.3–12.4]	64.8
Red marrow	5.4 [2.4–13.5]	3.8 [1.7–8.8]	29.6
Skeleton	9.1 [3.7–23.5]	5.0 [2.2–12.3]	45.1
Skin	6.6 [2.6–19.3]	4.4 [1.8–12.4]	33.3
Small intestine	10.1 [5.1–22.8]	9.8 [5.0–21.8]	3.0
Spleen	11.1 [5.4–25.4]	4.8 [1.9–15.1]	56.8
Stomach	11.6 [5.9–26.4]	7.0 [3.2–15.8]	39.7
Urinary bladder	11.7 [6.3–26.9]	11.0 [6.0–25.7]	6.0
Breasts	3.8 [0.5–18.8]	0.1 [0.1–0.6]	97.4
Ovaries	9.7 [5.8–19.4]	9.4 [5.7–18.8]	3.1
Uterus	9.7 [6.0–20.8]	9.5 [5.8–20.2]	2.1
Testicles	17.6 [5.4–52.9]	3.4 [0.7–32.7]	80.7

*p* ≤ 0.001; *mSv* Millisievert

### Image analysis

The entire appendix was visualized by full range and virtual reduced range CT in 81 of 90 patients while it was not detectable in 9 cases; in these patients, however, the whole caecum was depicted by full range and virtual reduced range CT. Based on the CT findings, appendicitis was present in 66 patients (73%) whereas an alternative diagnosis was made in 14 cases (16%). In 10 patients (11%), there were no distinct CT findings to explain the patient’s symptoms (Table 3).

**Table 3:** Final diagnosis based on CT findings

Diagnosis	No. (%)
Appendicitis	66 (73%)
Colitis/Enteritis	7 (8%)
Colitis/Enteritis with Diverticulitis	1 (1%)
Colitis/Enteritis with Abscess	1 (1%)
Pyelonephritis	2 (2%)
Cholecystitis	1 (1%)
Urolithiasis with ruptured renal cyst	1 (1%)
Abscess	1 (1%)
None (no findings explaining the patient’s symptoms)	10 (11%)

No appendicitis or differential diagnosis was missed by the virtual reduced range CT. Incidental findings which were not detected by reduced range CT are displayed in Table 4.

**Table 4:** Incidental findings not detected by virtual reduced range CT

Incidental finding	No.
Heart (CAD, pericardial effusion, thrombus)	19
Liver (Cystic lesion or hemangioma)	15
Lung (Pneumonia, dystelectasis, nodule)	11
Kidneys (Cystic lesion)	7
Adrenal gland (Adenoma)	4
Hiatal hernia	4
Spleen (Splenomegaly, cystic lesion)	2
Pancreas (Cystic lesion)	2
Pleural effusion	2
Steatosis hepatis	1
Kinking of the thoracic aorta	1
Hilar lymphadenopathy	1
Occlusion of the superficial femoral artery	1
Total	70

## Discussion

Our results indicate that a CT examination protocol with a limited scan range from the first lumbar vertebral body to the pubic symphysis reliably allows to confirm or to rule out acute appendicitis as well as differential diagnoses in patients with suspected acute appendicitis. Furthermore, this method is associated with a reduction of the whole body effective radiation dose of 39% alongside with a reduction of organ doses of up to more than 90% and can be applied independently of the used CT equipment.

**Fig. 3:**
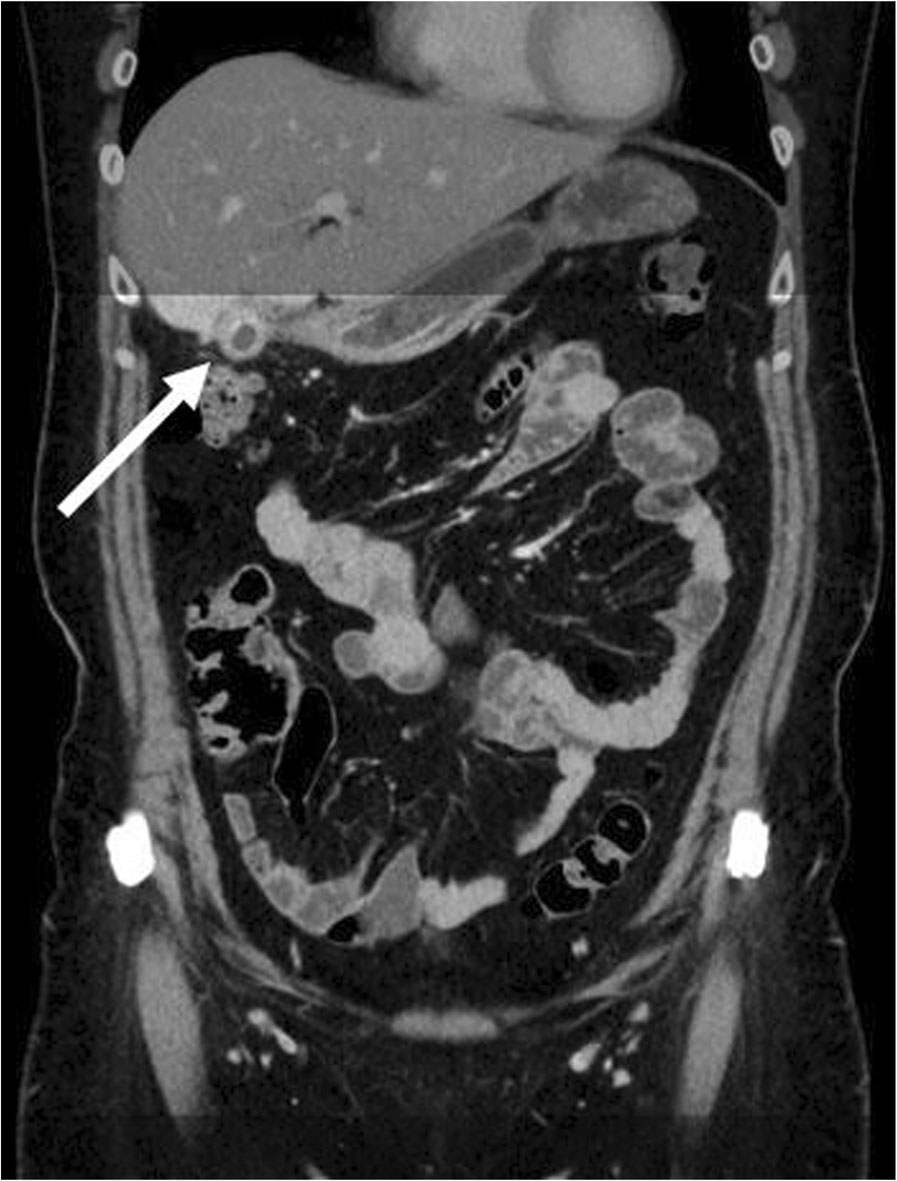
Coronal reformation of a CT examination of a female patient performed due to suspected acute appendicitis revealing acute cholecystitis, a finding which is marginally displayed by reduced range CT

**Fig. 4:**
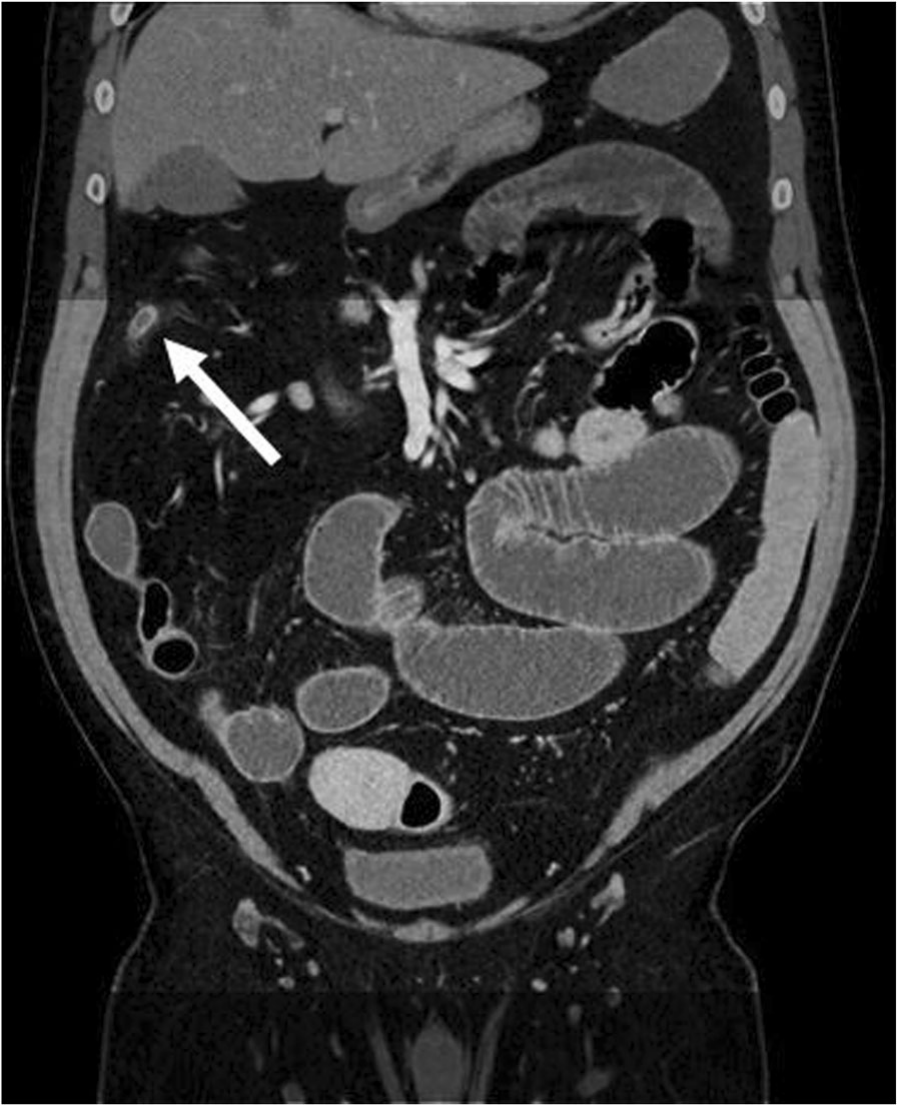
Coronal reformatted CT of a patient with acutely inflamed unusual high positioned appendix as an example for another marginally displayed finding in reduced range CT

Our study confirms the results of several previous studies investigating the approach of a CT examination limited not only to the pelvis, but to a certain anatomical area of the abdomen and pelvis as defined by bony landmarks on the localizer radiograph in these patients [12, 14]. As in these works, our limited CT examination depicted each case of acute appendicitis as well as differential diagnoses as compared to full range CT. However, in contrary to two of the mentioned studies which defined the top of the second lumbar vertebral body as upper border of the limited CT scan, we chose L1 as upper limit based on our clinical experience in order to visualize greater portions of the upper abdominal organs and therefore enable an improved detection of possible alternative diagnoses. As an example, CT revealed acute cholecystitis in one female patient in our cohort presenting with pain in the lower right quadrant, a finding that would not have been depicted with a scan starting at the top of L2 (Fig. 3). Another pitfall when the upper limit of the scan range is set too low is an atypical high position of the appendix as shown in another example of our cohort (Fig. 4). Therefore, we recommend to apply a limited CT protocol only in patients with high likelihood of acute appendicitis and in combination with an ultrasound examination of the upper abdominal organs, especially of the gall bladder.

Compared to full-range CT, 70 incidental findings would have been missed when applying our reduced-range CT protocol. However, most of these are clinically irrelevant and none of these findings would be expected to explain the symptoms attributed to acute appendicitis. A minority of these incidentally detected lesions may require further diagnostic work-up (e.g. pulmonary nodules, pneumonia, pericardial/pleural effusion, ventricular thrombus), but CT of the abdomen and pelvis is not the method of choice to evaluate such findings and therefore is not warranted.

Shortening the scan range according to our suggestion enables a median reduction of the whole body effective dose of 2.9 mSv (39%). The authors of prior studies investigated the impact of reduced scan range on radiation dose or extent of anatomic coverage, which is directly related to radiation exposure, and reported a reduction in between 23 and 46%, well in line with our results [10–14]. However, only a minority of studies provided data concerning potential reductions of organ doses, limited to the breast dose in female and the testicle dose in male subjects. In contrary, our work is the first to add a detailed overview of potential dose savings for numerous organs by exploiting the potential of a modern dose monitoring and tracking tool, with statistically significant high dose reductions particularly for adrenals (82%), esophagus (85%), heart (94%), liver (55%), lungs (92%), pancreas (65%), spleen (57%), breasts (97%) and testicles (81%). On the other hand, we observed only marginal, nevertheless statistically significant dose reductions for colon (7%), small intestine (3%), urinary bladder (6%), ovaries (3%) and uterus (2%). These findings reflect not only the reduced anatomic coverage as in other studies, but also the decreased scatter radiation, which is additionally taken into account when using the dose monitoring and tracking software. The ovaries may serve as an example: Although being completely included in the scanning volume of full range and reduced range CT, their organ dose is slightly but significantly reduced due to decreased scatter radiation when performing a reduced range CT.

MRI is another powerful option in order to confirm or rule out acute appendicitis without any radiation exposure. Yet, it is not the method of choice in the context of suspected appendicitis due to its higher costs and longer scan time compared to CT. Furthermore, MRI is often not constantly available even in large hospitals. Other circumstances that may prevent the use of MRI are contraindications like claustrophobia, pacemakers and certain metallic implants [2].

In our study, CT confirmed the diagnosis of acute appendicitis in most of the included cases while only a minority of patients presented with either another or no definite cause of their symptoms. This is inconsistent with the results of a precedent review including a large number of patients referred for CT due to suspected appendicitis in which alternative diagnoses were identified more frequently than acute appendicitis [16]. However, in contrast to our work, the patient cohort included in this study did not undergo sonography prior to CT which may rule out or raise the suspicion of common alternative diagnoses like urolithiasis, cholecystitis, small bowel obstruction or adnexal masses in females.

Our study has limitations. First, it is a retrospective single-center study with limited sample size. The included CT examinations were conducted on different scanners with different reconstruction methods (different strengths of iterative reconstruction or filtered back projection), and the used contrast agent protocols were not uniform as a minority of examinations were performed with rectal contrast or without intravenous contrast. However, we investigated the impact of a shorter scan range which is independent of the used CT protocol. Furthermore, we do not have surgical or clinical confirmation of the diagnoses made by CT, but this was not the purpose of our study as we did not examine the accuracy of CT in patients with acute abdominal pain which has been done before. Despite the fact that the use of CT may cause concern especially in younger patients due to the associated radiation exposure, our cohort with a mean age of about 57 years does not quite match this age group. However, we do not consider this as a limitation as our results do not depend on patients’ age and can be transferred to younger individuals as well.

## Conclusions

In conclusion, we consider a CT protocol with reduced scan range from the top of the first lumbar vertebral body to the pubic symphysis as accurate as full range CT to diagnose acute appendicitis or alternative diagnoses in patients with suspected acute appendicitis. This approach is associated with a reduction of the whole body effective dose of 39% and a reduction of organ doses of up to more than 90%.

## Abbreviations

ADMIRE: Advanced modeled iterative reconstruction
SAFIRE: Sinogram affirmed iterative reconstruction
